# *fabH* deletion increases DHA production in *Escherichia coli* expressing *Pfa* genes

**DOI:** 10.1186/s12934-018-0928-5

**Published:** 2018-06-08

**Authors:** Laura Giner-Robles, Beatriz Lázaro, Fernando de la Cruz, Gabriel Moncalián

**Affiliations:** Departamento de Biología Molecular, Universidad de Cantabria and Instituto de Biomedicina y Biotecnología de Cantabria (IBBTEC), CSIC-Universidad de Cantabria, C/ Albert Einstein 22, 39011 Santander, Spain

**Keywords:** Fatty acid synthesis, Polyunsaturated fatty acid, Docosahexaenoic acid, Cerulenin, FabH

## Abstract

**Background:**

Some marine bacteria, such as *Moritella marina*, produce the nutraceutical docosahexaenoic acid (DHA) thanks to a specific enzymatic complex called Pfa synthase. *Escherichia coli* heterologously expressing the *pfa* gene cluster from *M. marina* also produces DHA. The aim of this study was to find genetic or metabolic conditions to increase DHA production in *E. coli*.

**Results:**

First, we analysed the effect of the antibiotic cerulenin, showing that DHA production increased twofold. Then, we tested a series of single gene knockout mutations affecting fatty acid biosynthesis, in order to optimize the synthesis of DHA. The most effective mutant, *fabH*, showed a threefold increase compared to wild type strain. The combination of cerulenin inhibition and *fabH* deletion rendered a 6.5-fold improvement compared to control strain. Both strategies seem to have the same mechanism of action, in which fatty acid synthesis via the canonical pathway (fab pathway) is affected in its first catalytic step, which allows the substrates to be used by the heterologous pathway to synthesize DHA.

**Conclusions:**

DHA-producing *E. coli* strain that carries a *fabH* gene deletion boosts DHA production by tuning down the competing canonical biosynthesis pathway. Our approach can be used for optimization of DHA production in different organisms.

**Electronic supplementary material:**

The online version of this article (10.1186/s12934-018-0928-5) contains supplementary material, which is available to authorized users.

## Background

Consumption of long-chain polyunsaturated fatty acids of the omega-3 series (LC-PUFA3) has been associated to benefits for human health [1]. LC-PUFA3 are found in membrane phospholipids and are especially abundant in neuron and retina. LC-PUFA3 play a major impact during fetal development, as well as in Alzheimer’s disease progression [1]. Furthermore, they reduce chronic inflammation, which is the cause of many cardiovascular diseases [2]. Since only limited amounts of LC-PUFA3 are synthesized in humans from α-linolenic acid (the main LC-omega-3 FA in vegetables), the Food and Agriculture Organization [3] recommends a minimum intake of 250 mg LC-PUFA3 per day, while pregnant women intake must be 300 mg/day, of which 200 mg should be docosahexaenoic acid (DHA). However, the recommended daily intake is not reached in many countries, where consumption of fish and seafood is insufficient.

Currently, the main source of supplemented LC-PUFA3 is fish oil. Problems arise in the industrial production of LC-PUFA3, such as elevated purification costs, low stability of the molecules, undesirable odors, flavors and tastes, or the presence of a wide range of contaminants, including heavy metals. Furthermore, concerns about the sustainability of fisheries have increased the efforts towards alternative production sources [4, 5].

Besides fish, animals, plants, fungi and microalgae synthesize LC-PUFA3 from α-linolenic acid by consecutive elongation and desaturation reactions. However, thraustochytrids and a group of deep-sea bacteria are able to produce de novo LC-PUFA3 by a multi-enzymatic protein complex named polyunsaturated fatty acid synthase (Pfa) [6]. The mechanism has not been characterized in detail, but is thought to be similar to Type II polyketide synthases (PKS) and de novo fatty acid synthases (FAS). LC-PUFA3 synthesis begins by the condensation of a malonyl group bound to an acyl-carrier protein (ACP) with an acetyl-CoA. This reaction is carried out by a β-ketoacyl-ACP synthase domain (KS). Later, there are several cycles of reduction, dehydration, reduction and condensation, to produce a 20-carbon long chain with five double bonds (C20:5, eicosapentaenoic acid, EPA) or a 22-carbon long chain with six double bonds (C22:6, DHA) [7, 8]. Orikasa et al. [7] cloned the five essential genes for DHA production from marine bacteria *Moritella marina* into an *E. coli* expression vector. The resulting plasmid, named pDHA4 (Table 1), contains *M. marina pfaABCD* genes under control of native promoters, while *pfaE* is controlled by a T7 promoter. *E. coli* strain DH5α containing pDHA4 produced 3.7% DHA of total FAs after 96 h at 15 °C [7].

**Table 1:** List of *E. coli* strains and plasmids used in this work

	Relevant characteristics	References
Strains		
BW25113	F-, DE(araD-araB)567, lacZ4787(del)::rrnB-3, LAM-, rph-1, DE(rhaD-rhaB)568, hsdR514	[16]
BW27783	BW25113 DE(araFGH) Φ(∆araEp PCPA–araE)	[19]
Plasmids		
pDHA4	pSTV29 (Takara Corp.) carrying pfaABCDE from *M. marina* MP-1 (p15A OriV, ~ 10 copies, chloramphenicol resistance cassette) pfaABCD expression is controlled by native *M. marina* promoters, pfaE is controlled by T7 promoter	[7]

DHA production can be improved by treatment with sub-lethal concentrations of cerulenin. This antibiotic increased DHA production in *M. marina* [9], *Colwellia psycherythraea* [10], *Thraustrochytrium* sp. [11], and even in *E. coli* expressing the heterologous Pfa system [7, 12]. Pfa synthase is similar to Fatty Acid Synthase (FAS), responsible for short and medium chain fatty acid biosynthesis and uses the same substrates, malonyl-CoA and acetyl-CoA. In *E. coli*, de novo FAS-based FA synthesis uses three KS enzymes named FabB, FabF and FabH, which initiate FA elongation cycle. Although the three KS enzymes are structurally similar and participate in a similar reaction, the antibiotic cerulenin inhibits FabB and FabF, but not FabH [13, 14]. Thus, inhibition of FabB and FabF by cerulenin decreases the amount of substrate consumed by FAS, increasing by 50-fold the intracellular level of malonyl-CoA available for heterologous synthetic pathways [15].

In this article we tested single gene in-frame knock-outs in *E. coli* to find genetic modifications which improve the DHA production by Pfa heterologous system from *M. marina*. In particular, we found that *fabH* deletion produced a threefold improvement compared to the wild type reference strain. This modification could be used in oleaginous DHA-producing microbial species to enhance the production of this added-value fatty acid.

## Results

### Cerulenin enhances DHA production in *E. coli* expressing *M. marina pfa* gene cluster

The positive effect of the antibiotic cerulenin on the production of DHA in marine bacteria was previously known. DHA-producer *M. marina* treated with sub-lethal concentrations of cerulenin (0.5–2 µg/ml) showed a threefold increase (6–19%) in DHA production [9], while *Colwellia psycherythraea* treated with 12 µg/ml cerulenin showed a fourfold increase in DHA content, accumulating up to 10% of total FA [10]. However, little is known about genetic modifications enhancing this production.

To study genetic effects on DHA production we have used the KEIO collection [16], a collection of single-gene deletions of all nonessential genes in *E. coli* K-12. First, we transferred pDHA4 to *E. coli* strain BW27783 (BW-pDHA4 from here), a derivative strain from KEIO control strain BW25113 suitable for future assays with pBAD33 expression system, in which arabinose transporter and arabinose degradative enzymes have been modified [17–19]. BW-pDHA4 showed an increased production in DHA in 72 h when compared to *E. coli* DH5α reported by Orikasa et al. [7] (from 3.7 to 7.5% DHA of total FA).

We have first determined the optimal culture time and temperature for DHA production using BW-pDHA4 (Additional file 1: Fig. S1). We tested DHA production at different culture times obtaining 4.7% DHA/total FA at mid-exponential phase (24 h), 7.0% at the end of exponential phase (48 h), and 4.6–5.1% during stationary phase (up to 96 h). In addition, we tested DHA content at late exponential phase in cultures at different temperatures (7.1% DHA/total FA at 15 °C, 3.5% at 20 °C, and 0% at 25 °C). Therefore, we established that the best conditions to produce DHA using BW-pDHA4 strain were 48 h at 15 °C.

**Fig. 1:**
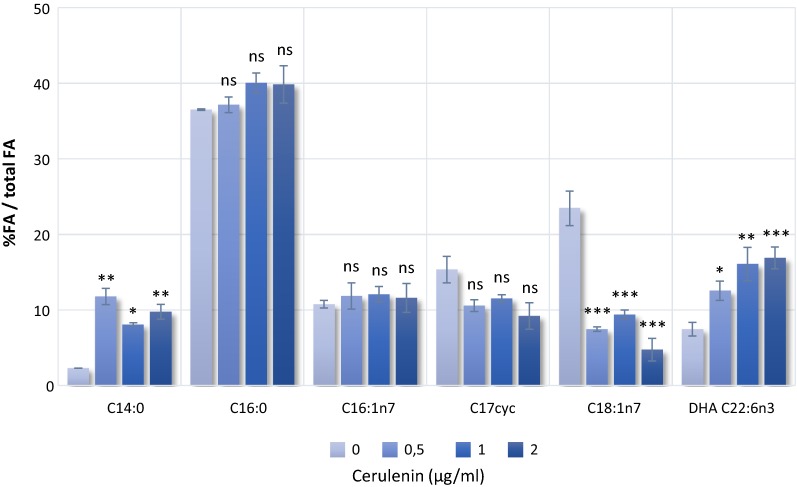
Composition of major fatty acids in *E. coli* BW 27783 (pDHA4) treated with different concentrations of cerulenin (0–2 µg/ml) at 15 °C for 72 h. Bars indicate the mean and standard deviation of the percentage of each FA in relation to total FA. Statistical analysis for each FA was made individually for at least three replicates using One-Way ANOVA. Asterisks indicate the degree of significance (***p < 0.0001; **p < 0.001; *p < 0.05; *ns* not significant p > 0.05). Miristic acid—C14:0, palmitic acid—C16:0, palmitoleic acid—C16:1, C17 cyclopropane derivative—C17cyc, cis-vaccenic acid—C18:1n7, DHA—C22:6n3

Afterwards, we studied the enhancement of DHA production in the presence of different concentrations of cerulenin. As shown in Fig. 1, DHA (C22:6n3) content in BW-pDHA4 increased from 7.5 to 12.5, 16.1 or 17.0% in the presence of 0.5, 1 or 2 µg/ml cerulenin, respectively. Even though BW-pDHA4 achieved the highest DHA content at a cerulenin concentration of 2 µg/ml, we performed the subsequent experiments using 1 µg/ml since the DHA (C22:6n3) content is almost similar at 1 or 2 µg/ml and we had observed that high concentrations of cerulenin affected cell growth (Additional file 1: Fig. S2).

Cerulenin also had an effect on the overall FA composition in BW-pDHA4 (Fig. 1). Medium-chain saturated fatty acid content such as miristic acid (C14:0) increases in presence of cerulenin, while levels of palmitic acid (C16:0), palmitoleic acid (C16:1) and C17 cyclopropane acid (C17cyc) have not significant variations. In contrast, cis-vaccenic acid (C18:1n7) content drastically decreases. Similar variations in the fatty acid profile were reported for *M. marina* [9], C. *psycherythraea* [10] or *E. coli* expressing *C. psycherythraea pfaABCDE* genes [12].

### DHA production is increased in Δ*fabH* mutant

**Fig. 2:**
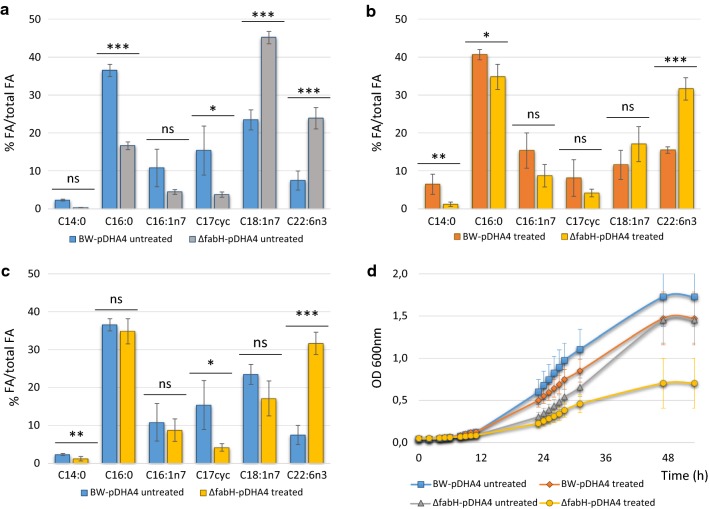
Effect of *fabH* deletion and cerulenin on heterologous DHA production in *E. coli* strains cultured at 15 °C for 72 h. **a** Effect of *fabH* deletion on fatty acid composition of BW-pDHA4 and ∆FabH-pDHA4 in absence of cerulenin (untreated strains). **b** Effect of cerulenin on BW-pDHA4 and ∆FabH-pDHA4 in presence of 1 μg/ml cerulenin (treated strains). **c** Additive effect of *fabH* deletion and cerulenin treatment on fatty acid composition. Fatty acids from ∆*fabH*-pDHA4 treated with 1 μg/ml cerulenin are compared to fatty acids of untreated BW-pDHA4. Mean and standard deviation of each FA in relation to total FA content is shown. Statistical analysis was made for at least three replicates using One-Way ANOVA. Asterisks indicate the degree of significance (***p < 0.0001; **p < 0.001; *p < 0.05; *ns* not significant p > 0.05). Miristic acid—C14:0, palmitic acid—C16:0, palmitoleic acid—C16:1, C17 cyclopropane derivative—C17cyc, cis-vaccenic acid—C18:1n7, DHA—C22:6n3. **d** Growth curve of BW-pDHA4 and ∆FabH-pDHA4 with 0 and 1 μg/ml of cerulenin. Each curve was measured for 52 h at 15 °C using 12 independent cultures

Cerulenin was shown to inhibit FabB and FabF, two main KS synthases involved in conventional FA synthesis [14]. Both enzymes are responsible for condensing malonyl-ACP with the FA growing chain. Besides FabB and FabF, FabH is the KS enzyme responsible of the initial condensation of malonyl-ACP to either acetyl-CoA or propionyl-CoA. However, FabH is not inhibited by cerulenin [20]. Thus, we hypothesized that, similar to the inhibition of FabF and FabB by cerulenin, deletion of *fabH* could also lead to an increase of DHA production. To test the *fabH* deletion phenotype, we transformed a *fabH* knockout mutant strain from the KEIO collection [16] with pDHA4 (ΔfabH-pDHA4 from here). In Fig. 2a, we can observe a substantial improvement of DHA production caused by *fabH* deletion, reaching 24% DHA relative to total FAs. This level of DHA production implies a threefold improvement compared to BW-pDHA4, and corresponds to 11.2 mg DHA/L (Table 2). Additionally, the absence of FabH activity altered the FA composition. Comparing the FA profile in Fig. 2a between BW-pDHA4 and ΔfabH-pDHA4 strains, we observe a major decrease in C16:0 (36.5% BW-pDHA4 vs 16.7% ΔfabH-pDHA4), and C17cyc (15.4% vs 3.8%). In contrast, there is a significant increase in long-chain FAs, such as C18:1n7 (23.5% vs 45.2%), as well as the already mentioned increment in DHA. Furthermore, *fabH* deletion had an impact on cell growth (Fig. 2d and Table 2). Cells carrying this deletion (ΔfabH-pDHA4) had a slower growth and thus a lower doubling time (D_T_, Table 2) than reference strain BW-pDHA4 (5.3 h in BW-pDHA4 vs. 7.2 h in ΔfabH-pDHA4).

**Table 2:** Effect of *fabH* deletion and cerulenin inhibition (1 µg/ml) on DHA producing strains

Strain	BW—pDHA4		ΔfabH—pDHA4	
Cerulenin	0 µg/ml	1 µg/ml	0 µg/ml	1 µg/ml
Doubling time (h)^a^	5.3 ± 0.2	6.2 ± 0.2***	7.2 ± 0.2***	8.8 ± 0.7***
%DHA/total FA^b^	7.5%	15.5%**	23.9%***	31.7%***
mg DHA/l^b^	2.8 ± 0.4	8.1 ± 0.24***	11.2 ± 1.9***	16.8 ± 1.6***

^a^Maximum doubling time calculated during the exponential phase for each condition

^b^DHA production is represented by relative (% DHA/total FA) and absolute (mg DHA per liter of culture) quantification of DHA for each strain and condition

Mean and standard deviation is shown. Asterisks indicate the degree of significance (*** p < 0.0001; ** p < 0.001)

In order to observe the effect of a tighter inhibition on the system, we analysed the effect of cerulenin on ΔfabH-pDHA4 cells. In this experiment, FA production was altered simultaneously by *fabH* deletion and by FabB and FabF partial inhibition with cerulenin. From the data in Fig. 2b, we can see that, under these doubly limiting conditions, DHA production reached 31.7% of total FAs (16.8 mg DHA/L), 4.9-fold higher than BW-pDHA untreated. The proportion of some other FA species in ΔfabH-pDHA4 treated with cerulenin also changed compared to untreated BW-pDHA (Fig. 2c). For instance, the proportion of miristic acid in ΔfabH-pDHA4 treated with cerulenin (2.3%) is significantly lower than in treated ΔfabH-pDHA4 (1.2%) while C17cyc is reduced from 15.4% in untreated BW-pDHA4 to 4.2% in treated ΔfabH-pDHA4. In relation to cell growth, this double inhibited system showed a low growth (Fig. 2d) with a D_T_ of 8.8 h (Table 2).

### Effect of other KO mutants on DHA production

**Fig. 3:**
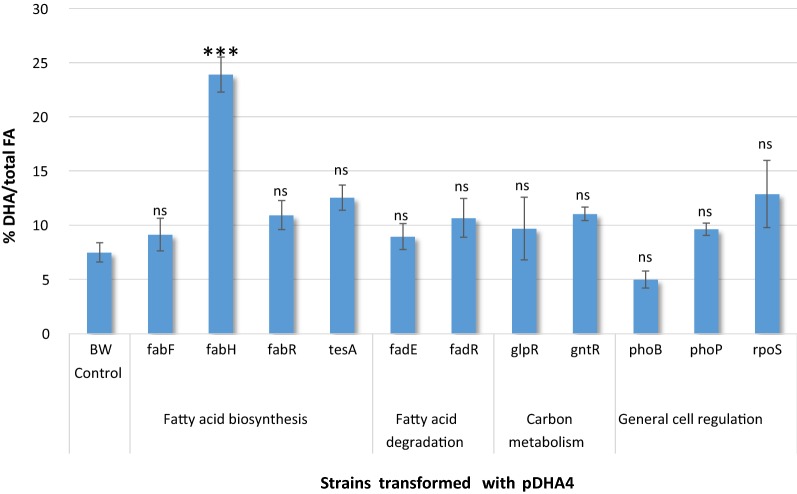
DHA production in KEIO mutant strains and reference strain BW27783 transformed with pDHA4 plasmid. Bars indicate the mean and standard deviation of DHA percentage in relation to total FA content. Statistical analysis of each FA content for each strain was performed for at least three replicates using One-Way Anova. Asterisks indicate the degree of significance (***p < 0.0001; *ns* not significant p > 0.05)

To check the effect of other FA-synthesis mutations of the KEIO collection, as well as to stablish a control for the effect of in-frame deletions, we tested DHA production in a series of KEIO mutants transformed with pDHA4 (Fig. 3). The following mutants were selected (Table 3):

**Table 3:** Selected mutants from KEIO collection [16] for improvement of DHA production by expression of pDHA4 plasmid

Gene name	Gene code	Protein	Process
*fabF*	b1095	Ketoacyl synthase II	Fatty acid biosynthesis
*fabH*	b1091	Ketoacyl synthase III	
*fabR*	b3963	Fatty acid synthesis repressor	
*tesA*	b0494	Acyl-CoA thioesterase I	
*fadE*	b0221	Acyl-CoA dehydrogenase	Fatty acid degradation
*fadR*	b1187	Global lipid metabolism regulator	
*glpR*	b3423	Glycerol-3-phosphate transcriptional repressor	Carbon metabolism regulation
*gntR*	b3438	Entner-Doudoroff pathway transcriptional repressor	
*phoB*	b0399	Response factor. Phosphate dual regulation factor	General cell regulation
*phoP*	b1130	Response factor. Phosphate dual regulation factor	
*rpoS*	b2741	Sigma factor. Represses of *fadA*, *fadB*, *fadE* and *fadH*	

Gene code is taken from *E. coli* MG1655 strain in Ecocyc database

i. Three non-essential genes related to FA biosynthesis besides *fabH*: *fabF* (ketosynthase responsible for cis-vaccenic production), *fabR* (global regulator of fatty acid metabolism) and *tesA* (FA thioesterase). These three mutant strains, transformed with pDHA4, did not show a significant improvement in DHA production compared to control strain BW-pDHA4. As shown in Fig. 3, DHA content increased from 7.5% in BW-pDHA4 to 10.9% in Δ*fabR*-pDHA4 and 12.5% in Δ*tesA*-pDHA4 under the same culture conditions. Curiously, the single deletion of *fabF* (ΔfabF-pDHA4) only increased DHA production to 9.1%, a less prominent effect than the dual partial inhibition of FabB and FabF produced by cerulenin (17%) shown previously.
ii. Two genes coding for enzymes involved in the FA degradation pathway, in order to avoid DHA consumption: *fadE* (FA β-oxidation enzyme), *fadR* (FA metabolism regulator). None of these two mutants showed a significant difference in the DHA content compared to BW-pDHA4, since Δ*fadE*-pDHA4 and Δ*fadR*-pDHA4 had a DHA content of 8.9 and 10.7% respectively.
iii. Deletion of gene regulation factors involved in carbon metabolism pathways: *glpR* (repressor of the glycerol-3-phosphate regulon) and *gntR* (repressor of the Entner-Doudoroff pathway). Both mutants did not have a significant difference in DHA content compared to the BW-pDHA4, from 7.5 to 9.8% in Δ*glpR*-pDHA4 and 11.0% in Δ*gntR*-pDHA4.
iv. Three genes coding for general regulation proteins involved in the FA metabolic switch between biosynthesis and degradation [20]: *phoB* and *phoP* (dual regulator involved in FA regulation network) and *rpoS* (sigma factor involved in FA regulation network). The regulation mutants did not show a significant difference in the DHA content compared to BW-pDHA4, since Δ*phoB*-pDHA4, Δ*phoP*-pDHA4 and Δ*rpoS*-pDHA4 had a 5.0, 9.6 and 12.9% of DHA.

## Discussion

The main goal in this work was to obtain an *E. coli* strain with the ability to produce higher amounts of DHA, avoiding the addition of exogenous expensive compounds such as cerulenin to the culture. Previous studies [9–11] suggested that there is substrate competition between FAS and Pfa biosynthesis pathways, since Claisen condensation of malonyl-ACP and acetyl-CoA initiates both synthetic pathways. Sublethal concentrations of cerulenin partially alter the KS activity of FabB and FabF in FAS system, which increases the concentration of the intracellular malonyl-CoA pool available for the heterologous DHA pathway [15]. Furthermore, it has been previously reported [11] that the heterologous Pfa system from *Thraustochytrium* sp. expressed in *E. coli* was not inhibited by cerulenin, while the FAS system was indeed affected. Thus, the Pfa pathway seems to use the intracellular malonyl-CoA pool for DHA production, which increases when conventional FA synthesis (via the *fab* pathway) is partially blocked.

**Fig. 4:**
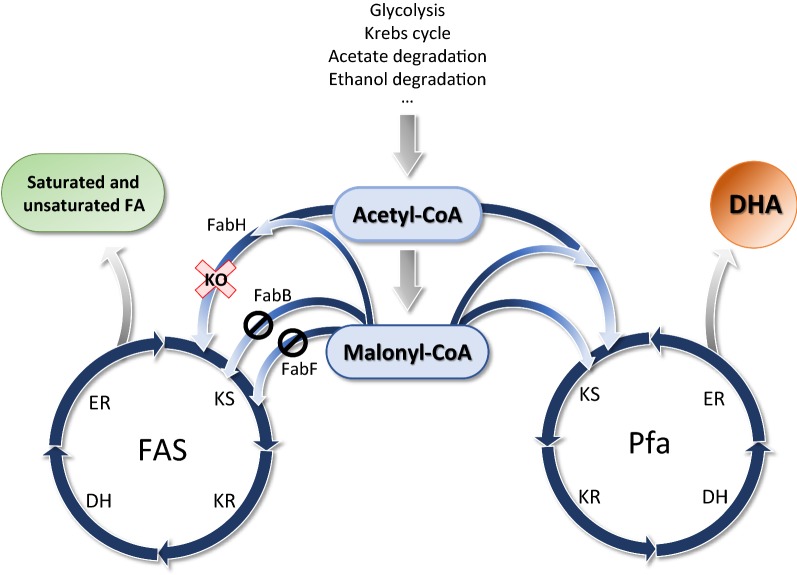
Scheme that represents a model for metabolic utilization of acetyl-CoA and malonyl-CoA by FAS and Pfa pathways. In a cell carrying the Pfa system, there is competition for substrate, acetyl-CoA and malonyl-CoA between Pfa and FAS biosynthetic pathways. Malonyl-CoA is mostly consumed by FAS synthase for canonical FA production. Partial inhibition of the initial step of FAS activity either by gene deletion (KO, red cross) or cerulenin enzymatic inhibition (block sign) increases DHA production due to lower consumption of the substrates by FAS, which accumulate in the cytoplasm and can be redirected to the alternative Pfa pathway. *KS* ketosynthesis, *KR* ketoreduction, *DH* dehydration, *ER* enoylreduction

In fact, our in vivo results showed that DHA production is certainly improved in strain Δ*fabH*-pDHA4 (Fig. 2), even when cerulenin is not present in the medium, which could indicate that the availability of malonyl-CoA is actually a bottleneck for DHA production in our system. Thus, as shown in the model we present in Fig. 4, our data suggests the existence of a substrate competition mechanism between the FA synthase systems FAS and Pfa, analogous to what happens in the case of cerulenin inhibition.

In fatty acid synthesis, substrates bound to ACP are processed by three different KS. In the first cycle of elongation, FabH (KS III) condenses the substrates malonyl-ACP and acetyl-CoA to produce acetoacetyl-ACP. Afterwards, FabB (KS I) plays the essential role of elongating the primer acetoacetyl-ACP to 16-C long FA and elongate unsaturated fatty acids. Furthermore, in vitro studies found that FabB and FabF are also capable of using acetyl-ACP as a substrate by a side reaction in which malonyl-ACP is decarboxylated to acetyl-ACP, and then used for Claisen condensation with a malonyl-ACP residue [21]. Although this hypothesis is based on an in vitro assay, it could explain the residual FA synthetic activity in the strain carrying *fabH* deletion. Furthermore, it has been observed that a Δ*fabH Lactobacillus lactis* strain still retained 10% FA activity [22], probably due to side initiation KS reactions carried out by FabB and FabF [23].

Finally, last elongation cycle in *E. coli* is catalysed by FabF (KS II), adding two carbons to palmitoleic acid to produce cis-vaccenic acid [20]. This subsequent activity of the three ketosynthases could explain why the deletion of the main initial condensation enzyme of FA biosynthesis seems to have an even larger impact on DHA production than the inhibition caused by cerulenin, since FabH catalyses an earlier step during biosynthesis than FabB and FabF. Furthermore, the secondary catalytic ability of FabB is observed in Δ*fabH* strain treated with cerulenin, which shows a higher level of DHA, meaning the levels of intracellular malonyl-CoA is even higher.

Additionally, FabH has been reported as essential in strains lacking *spoT* [22]; however, in wild type strains, it has been proven to not be essential [16, 24]. As showed in Fig. 2c, the deletion of *fabH* has an impact on cell growth, same way the inhibition of cerulenin on FabB/FabF activity does.

Interestingly, deletion of *fabF* did not have a noteworthy effect on DHA content. This fact could be explained by the subsequent activity of the ketosynthase enzymes explained previously, since the cell is able to generate long-chain FAs by FabB activity, and therefore, FabF activity is not essential. In summary, these data indicate that, in order to increase the intracellular malonyl-CoA pool, it is necessary to intercept the metabolic route at the initial condensation steps of FA synthesis, or even earlier, at acetyl-CoA carboxilase (ACC) or FabD reactions. However, FabH is the only non-essential gene at this stage of FA synthesis, making it a good starting point for malonyl-CoA intracellular accumulation. Further analysis of other KEIO mutants shows that genes implicated in the regulation of FA metabolism had no significant improvement in DHA production. First, the transcription factors FabR, FadR and RpoS, as well as PhoB and PhoP, coordinate the biosynthesis and degradation of FA according to the energetic requirements of the cell [20]. It would be expected that elimination of the positive regulators of FA biosynthesis led to a significant increase of DHA. Nevertheless, no improvement in DHA production was observed. Thus, we could only speculate that the tight regulation of the FA metabolic pathways is exerted by modulation of the key enzymes of the cycle in order to respond to the energetic status of the cell [25].

Furthermore, we selected the enzyme from β-oxidation pathway FadE, with the aim of reducing FA degradation and, thus, avoiding DHA catabolism. However, this deletion had no impact on DHA accumulation. This observation was shocking since several studies reported that deletion of *fadE* and other genes of the β-oxidation pathway do reduce FA degradation, and therefore, increase FA accumulation [20].

## Conclusions

The main conclusion of this work is that deletion of *fabH* in a LC-PUFA3 producer organism, which carries a Pfa synthase, improves DHA production by diminishing substrate competition with FAS. Furthermore, this approach could be applied to other metabolic routes, which share malonyl-CoA as common precursor with FA synthesis, such as polyketide synthases, and other metabolites.

This approximation is a proof of concept that shows the potential of altering carbon fluxes towards a heterologous pathway.

## Methods

### Bacterial cells and cultivation

*Escherichia coli* BW27783 (Table 1) was transformed with plasmid pDHA4 [7] to be used as host for the recombinant *pfa* genes. Bacterial cells were inoculated from an overnight culture at 37 °C grown to saturation. Cultures at an initial OD = 0.15 were incubated on a rotary shaker (170 rpm) at 15 °C for 72 h in LB medium (Conda laboratories). When appropriate, kanamycin (50 µg/ml, Apollo Scientific) or chloramphenicol (25 µg/ml, Apollo Scientific) were added to the medium. In order to test the effect of cerulenin on cells, cultures were treated with a range of cerulenin concentrations (0–2 µg/ml, Cayman chemicals). Growth was determined by measuring the optical density at 600 nm (OD600) in a spectrophotometer (Nanodrop2000c, Nanodrop). Cells were collected by centrifugation in a Eppendorff 5810R centrifuge at 1700×*g* for 10 min at room temperature.

The growth curve of each strain was measured by OD at 600 nm in a Victor 3 system (Perkin Elmer) using 24-well plates with a culture volume of 750 µl. Each data point for the curve was calculated by the average of 12 independent cultures, and the maximum doubling time was calculated at early exponential phase.

Selected in frame mutants from KEIO collection [16] are summarized in Table 3. The strains were transformed with plasmid pDHA4 by electroporation, and checked for chloramphenicol resistance on LB agar plates (1.5% (wt/v) agar; Conda laboratories) Gene deletion of *fabH* was checked by PCR using primers FabHS (5′-GCTACAAAAGAGCCTGACGAGG) and FabHE (5′-CAATCACACCAGCGCAAACCAG), and primers for kanamycin resistance insertion Rk1 (5′-AGCCGAATAGCCTCTCCACCCA) and Fk2 (5′-CATCTCACCTTGCTCCTGCCGA) as described previously [18].

### Fatty acid analysis

FA composition and DHA quantitation were carried out by gas chromatography at Biomar Microbial Technologies (León, Spain) using pelleted cells. FAs were isolated from the samples following the Folch method [26]. The esterification with methanol was performed as described previously [27]. Concentrated FAMEs were analyzed on a gas chromatograph (model 7890A, Agilent) equipped with a capillary column DB-23 and flame ionization detection using helium as a carrier gas at 52.248 psi constant pressure. Temperature was kept at 130 °C for 1 min, then it was increased to 215 °C at a 2.75 °C/min ratio, and finally it was maintained at 215 °C for 12 min. The column was then cleaned by a ballistic increase of temperature to 230 °C and kept at this temperature for 2 min. The sample was injected at 270 °C in a split–splitless chamber with a split ratio of 10:1. The compounds were detected in a flame ionization detector (FID) at 280 °C with the following gas flows: air (200 ml/min), H_2_ (30 ml/min), and N_2_ (22.5 ml/min). FAMEs were identified by comparing the retention time with commercial standard Supelco37 Component FAME Mix (Sigma Aldrich). Quantitation of DHA was determined using commercial standards. Methyl tricosanoate was used as internal standard.

### Statistical analysis

The statistical analysis of the gas chromatography results was made using IBM SPSS Statistics version 22.0. (IBM Corporation). Comparison of group means against the control group was performed individually for each FA by the One-Way ANOVA software using Dunnet post hoc analysis. The significance value was set at 0.05.

## Additional file

**Additional file 1.** Additional Figures S1 and S2.
