# Supplementary material for: fabH deletion increases DHA production in Escherichia coli expressing Pfa genes
Source: Microb Cell Fact. 2018 Jun 8;17:88. doi: 10.1186/s12934-018-0928-5 (PMC5992819; doi:10.1186/s12934-018-0928-5)
Supplement: Supplementary file 1 — Additional file 1. Additional Figures S1 and S2. [file 12934_2018_928_MOESM1_ESM.pdf]

fabH deletion increases DHA production in *Escherichia coli* expressing *Pfa* genes

Laura Giner-Robles, Beatriz Lázaro, Fernando de la Cruz* and Gabriel Moncalián*

Supplementary Figures:

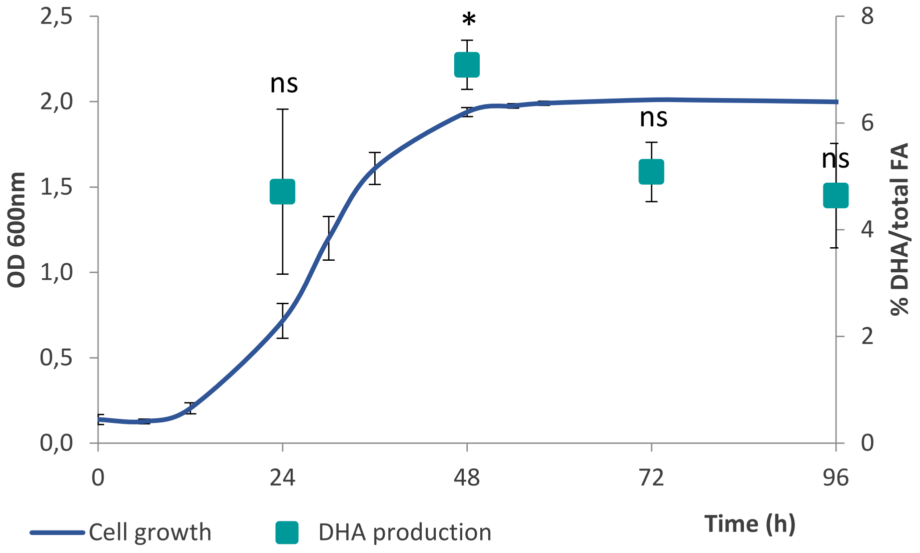

Figure S1. Kinetics of growth (line) and DHA production (squares) in BW-pDHA4 at 15°C for 96 h in three independent cultures. Bars indicate the mean and standard deviation. Asterisks indicate the degree of significance in the difference of DHA content among the samples (* $p<0.05$; ns - not significant $p>0.05$).

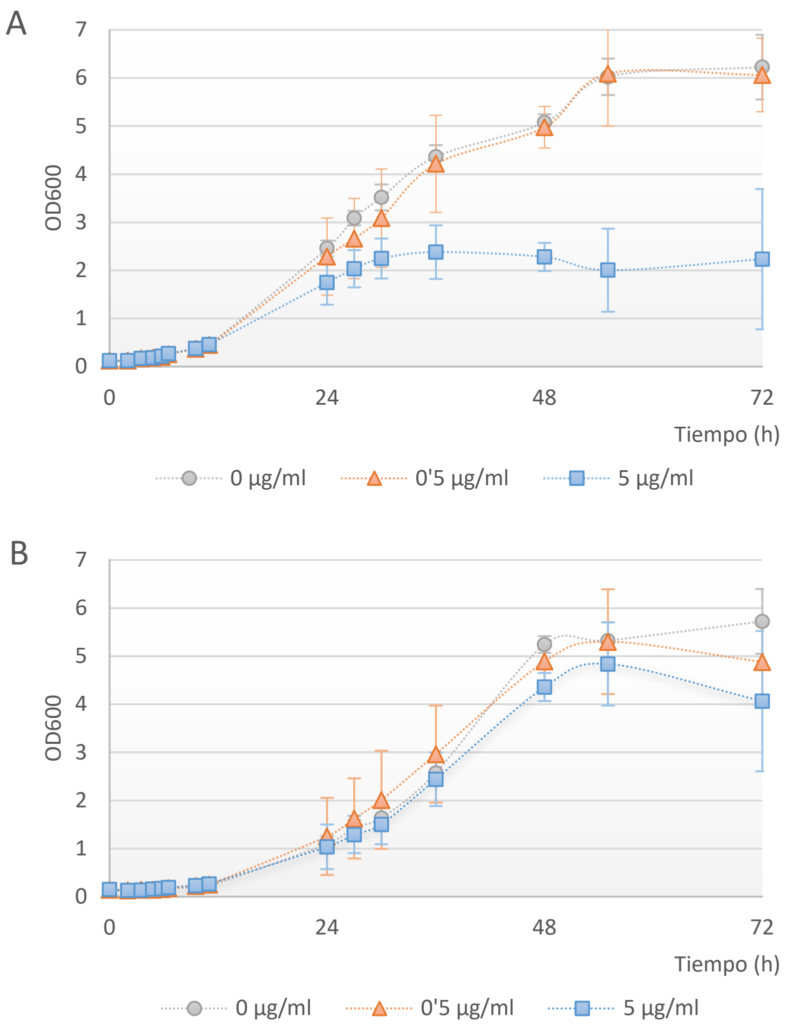

Figure S2. Growth curves of *E.coli* BW27783 (A) and *E.coli* BW27783 carrying plasmid pDHA4 (B) in absence of cerulenin (grey circles, 0 µg/ml), low concentration of cerulenin (orange triangle; 0.5 µg/ml), and high concentration of cerulenin (blue square; 5 µg/ml) for 72 h at 15 °C using 3 independent cultures. Mean and standard deviation is shown.
